# Leaf length-to-width ratio links non-destructive area estimation to functional trait variation in the pachymorph bamboo *Neosinocalamus affinis* across subtropical China

**DOI:** 10.1038/s41598-026-53448-w

**Published:** 2026-08-07

**Authors:** Miao Liu, Chunju Cai, Guanglu Liu, Xiaopeng Shi, Shuguang Li, Shaohui Fan

**Affiliations:** 1 International Centre for Bamboo and Rattan, Beijing, 100102 China; 2 Key Laboratory of National Forestry and Grassland Administration for Bamboo & Rattan Science and Technology, Beijing, 100102 China

**Keywords:** Bamboo, Leaf economics spectrum, Leaf functional traits, Morphological classification, Non-destructive estimation, *Neosinocalamus affinis*, Specific leaf area, Stoichiometry, Ecology, Ecology, Plant sciences

## Abstract

The leaf length-to-width (L/W) ratio is a simple morphological descriptor that may integrate information about leaf allometry, hydraulic architecture and resource-use strategy, yet its potential to simultaneously improve non-destructive leaf-area estimation and diagnose leaf economics spectrum (LES) position within a single species is rarely tested. We investigated whether L/W ratio captures coordinated variation in leaf morphology, chemistry and photosynthetic performance in *Neosinocalamus affinis* (Rendle) Keng f. (*N. affinis*), a sympodial pachymorph bamboo with notably narrow, lanceolate leaves that is economically and ecologically important in subtropical China. We analysed 162 site-level observations of 14 leaf functional traits — leaf area (LA), leaf length (L), leaf width (W), leaf thickness (LT), specific leaf area (SLA), leaf dry matter content (LDMC), SPAD chlorophyll index, maximum photochemical efficiency (*F*v/*F*m), and leaf carbon (TC), nitrogen (TN) and phosphorus (TP) concentrations with their stoichiometric ratios — collected from 38 county-level sites in seven provinces of southern China. *k*-means cluster analysis on L/W identified three data-driven shape classes — broader (L/W ≤ 6.05, *n* = 65), elliptic (6.05 < L/W ≤ 7.18, *n* = 58) and slender (L/W > 7.18, *n* = 39) — and a class-specific multiple linear regression (MLR) model had the best predictive performance (*R*² = 0.937, RMSE = 1.72 cm², AIC = 196.6), outperforming pooled Montgomery, pooled MLR and class-specific Montgomery models. The Montgomery proportionality coefficient (*k*) increased monotonically from broader (0.598) through elliptic (0.636) to slender leaves (0.663). One-way ANOVA with Tukey HSD detected significant differences among shape classes for LA, L, W, LT, LDMC, TC, TN and TP (all *p* < 0.05), but not for SLA, SPAD or Fv/Fm. A strong SLA–LDMC trade-off (*r* = − 0.850, *p* < 0.001) and N–P coordination (*r* = 0.590, *p* < 0.001) were observed, whereas L/W and LA were only weakly related to chemical traits. Environmental variables explained limited trait variation (multiple-regression *R*² ≤ 0.15), and variance partitioning showed that 30–64% of trait variance occurred within sites. L/W-based classification provides a parsimonious framework that improves non-destructive leaf-area estimation in *N. affinis* while capturing part — but not all — of the intraspecific LES. Structural and dry-matter traits align with leaf shape, whereas SLA and photosynthetic performance are largely decoupled from morphology. The combined morphological and functional framework offers a practical tool for bamboo ecosystem assessment and clarifies how the LES manifests within a single morphologically plastic species.

## Introduction

Leaves are the primary interface for photosynthesis, transpiration and gas exchange, and their morphological and chemical traits jointly determine how plants acquire, use and retain resources^1–4^. Leaf area (LA) scales directly with whole-plant light interception and carbon assimilation, and has therefore been the focus of numerous non-destructive estimation methods based on easily measured linear dimensions such as leaf length (L) and leaf width (W)^5–11^. At the same time, LA does not function in isolation: it is coordinated with structural, chemical and physiological traits along the leaf economics spectrum (LES), a global framework first articulated by Wright et al.^1^ that describes the trade-off between fast-return strategies (high SLA, high leaf nitrogen concentration, low leaf dry matter content) and slow-return strategies (low SLA, low leaf nitrogen, high leaf dry matter content)^1–4,12–15^. Understanding how simple morphological descriptors connect to this broader functional-trait network is essential for predicting ecophysiological strategies and for interpreting how trait covariance is organised within species and across scales^16–22^.

The Montgomery equation, LA = *k* · L · W, is among the most widely used non-destructive leaf-area estimators^5,7–11^. Here LA is leaf area (cm²), L is leaf length along the midrib (cm), W is the maximum leaf width perpendicular to the midrib (cm), and *k* is a dimensionless proportionality coefficient that captures the fraction of the bounding rectangle occupied by the leaf lamina. The equation assumes a single, shape-independent *k*, an assumption that has been shown to break down in species with strong morphological plasticity^7–11,23,24^. Classified-fitting approaches that stratify leaves by shape before estimating *k* have improved predictive accuracy across multiple taxa, including bamboos^6–11,23–25^, but whether the classes that improve area estimation also correspond to meaningful axes of the LES has not, to our knowledge, been tested explicitly at the intraspecific level.

The leaf L/W ratio is a simple, scale-free descriptor of leaf shape that has been linked to several ecophysiological functions^20,21,26,27^. Narrower leaves have shorter distances from midrib to margin, which reduces the thickness of the leaf boundary layer and enhances convective heat and gas exchange, potentially improving thermal regulation under high-radiation conditions^20,28^. Narrower leaves may also have shorter hydraulic path lengths from midrib to leaf margin, affecting hydraulic conductance and vulnerability to embolism^29,30^. Leaf shape additionally covaries with support investment, venation, and leaf-mass-per-area-related anatomy in ways that can influence physiological differentiation^21,31–35^. Because L/W is independent of overall leaf size, it provides a promising axis along which to probe whether morphology, chemistry and photosynthetic performance co-vary within a morphologically plastic species.

Bamboos are ecologically and economically important across tropical and subtropical regions, including through carbon sequestration and rural livelihoods^36^. Although bamboos contribute substantially to global forest biomass and ecosystem services, their representation in trait-based plant ecology has lagged behind that of broadleaved trees and grasses, and the literature on bamboo functional traits has expanded only recently. Sun et al.^37^ showed that, across subtropical bamboo forests, leaf morphology and nutrient content jointly drive variation in leaf carbon-capture and economic traits. Liu et al.^38^ documented variations and trade-offs in leaf and culm functional traits across 77 woody bamboo species, demonstrating that LES-like trade-offs operate at the among-species level in the Bambusoideae. Guo et al.^39^ further showed that, in moso bamboo (*Phyllostachys edulis*), leaf stomatal traits regulate stand-level gross primary productivity. Within bamboos, however, most published work has focused on monopodial taxa (especially *Phyllostachys* species), whereas sympodial pachymorph bamboos remain markedly under-represented despite their dominance in south-western Chinese forests and agroforestry systems. *Neosinocalamus affinis* (Rendle) Keng f., commonly known as “Cizhu”, is one of the most important sympodial bamboos in south-western China, distributed across Sichuan, Chongqing, Guizhou and Yunnan, where it provides culm material for pulping and weaving and supports the livelihoods of rural communities^36,38^. Its characteristically narrow lanceolate leaves vary widely in size and L/W ratio, making it a suitable model system for probing intraspecific structure–function relationships. Yet to date, no study has tested whether morphological stratification of *N. affinis* leaves provides both a quantitative framework for non-destructive area estimation and a diagnostic of intraspecific LES position. The present work addresses this gap.

Building on this framework, we addressed three central questions. First, does stratification by L/W ratio improve the accuracy of non-destructive leaf-area estimation compared with pooled models? Second, do L/W-defined shape categories differ in structural, chemical and photosynthetic leaf traits? Third, what environmental and hierarchical sources explain variation in leaf functional traits across sites? We expected that shape-specific models would outperform pooled models because the proportionality coefficient between LA and L × W should vary with lamina shape. We further expected that leaf shape categories would differ more strongly in structural and dry-matter-related traits than in instantaneous photosynthetic indicators. Finally, because environmental variables were measured or assigned at the site level, we expected broad climatic variables to explain only part of the observed trait variation, with a substantial fraction attributable to within-site variation.

## Methods

### Study species and sampling design

**Fig. 1:**
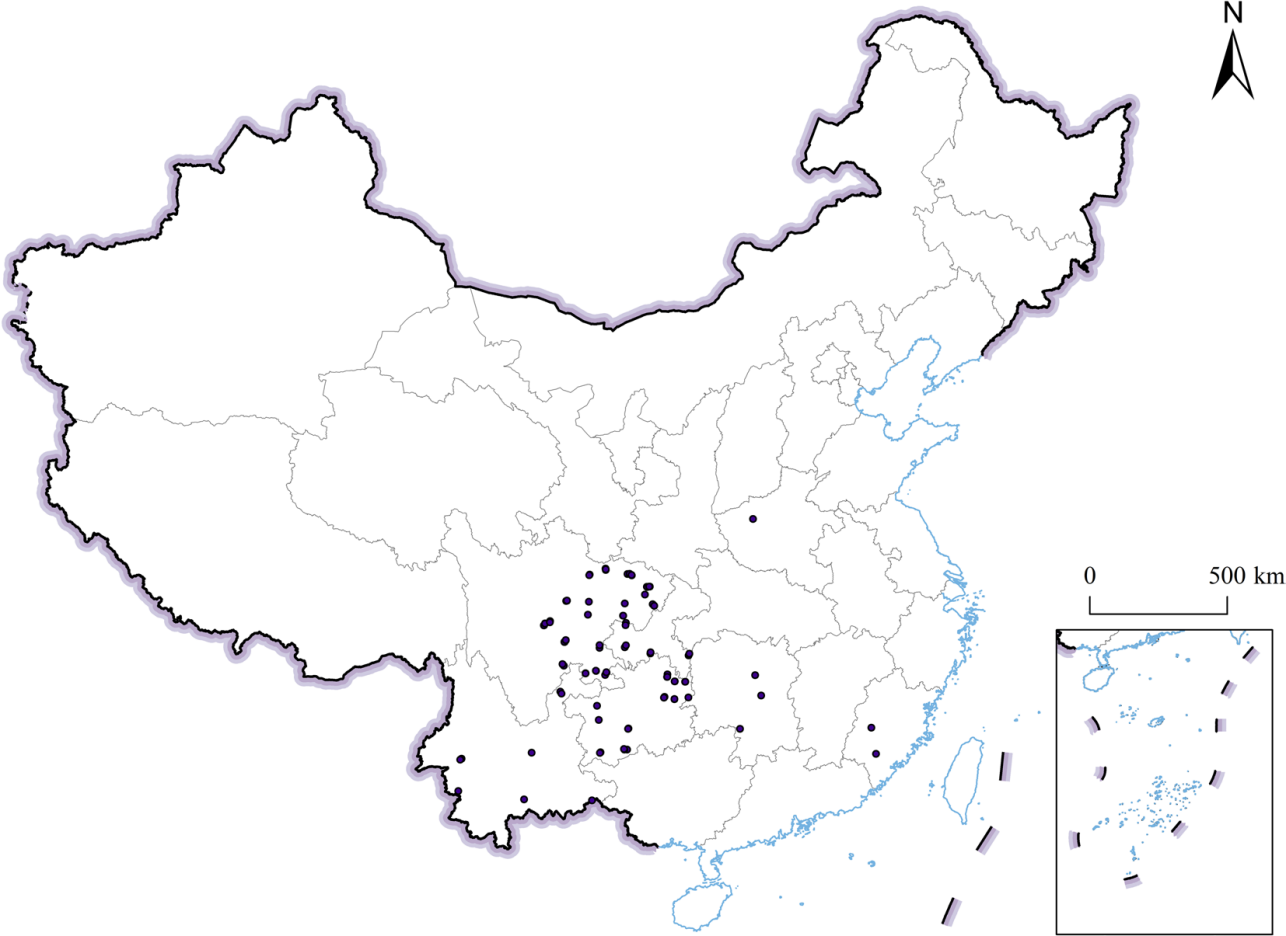
Geographic distribution of the 38 county-level sampling sites of *N. affinis* across seven provinces in southern China. Sites span 37–1,854 m in elevation, 9.9–20.6 °C MAT and 651–1,559 mm MAP.

*Neosinocalamus affinis* is a sympodial, pachymorph bamboo reaching 5–10 m in height, with narrow lanceolate leaves 8–20 cm long and 1–3 cm wide. Leaves of *N. affinis* were collected from 38 county-level sites distributed across seven provinces in southern China (Chongqing, Fujian, Guizhou, Henan, Hunan, Sichuan and Yunnan) during the 2023 and 2024 growing seasons (May–September; Table 1; Fig. 1). Sites spanned 37–1,854 m in elevation, 9.9–20.6 °C in mean annual temperature (MAT) and 651–1,559 mm in mean annual precipitation (MAP), covering most of the climatic envelope of the species.

At each site, 3–8 mature, healthy bamboo clumps were randomly selected at least 10 m apart to minimise clonal dependence, avoiding edge individuals, recently disturbed stands and clumps showing visible signs of pest damage or nutrient deficiency. From each clump, fully expanded, sun-exposed leaves were sampled from current-year culms (2–3 years old) at mid-canopy height (approximately 2/3 of total height) on south-facing branches to standardise light environment. Only fully expanded, non-senescent leaves from the middle portion of leafy branches were retained. Within each clump, 3–10 leaves were pooled to form a clump-level sample, and 2–8 clump-level samples were averaged to produce the site-level observations analysed here. This procedure yielded 162 site-level observations across the 38 sites. Plant material was formally identified by Miao Liu (co-author) at the International Center for Bamboo and Rattan, Beijing. Voucher specimens were deposited in the herbarium of the Key Laboratory of National Forestry and Grassland Administration for Bamboo & Rattan Science and Technology (herbarium code: ICBR; voucher numbers ICBR-Neosinocalamus-2023001 to 2023038).

**Table 1:** Summary of the 38 county-level sampling sites of *N. affinis* in southern China. n = number of site-level observations per county. LA = leaf area. Values are means ± SD; “—” indicates that SD is not defined (*n* = 1).

Province	County	Coordinates (°E, °*N*)	Elevation (m)	*n*	Mean LA (cm²)
Chongqing	Wulong	107.45, 29.18	252–942	5	29.4 ± 4.7
Chongqing	Yubei	106.30, 29.41	173–230	2	27.3 ± 0.5
Fujian	Huaan	117.32, 25.00	218	1	22.3 ± —
Fujian	Yongan	117.20, 26.04	229	1	22.8 ± —
Guizhou	Bijiang	108.94, 27.43	419–438	5	16.9 ± 7.3
Guizhou	Chishui	105.47, 28.31	343–499	6	22.6 ± 6.0
Guizhou	Longli	106.56, 26.19	1235–1266	6	16.2 ± 5.1
Guizhou	Luodian	106.46, 25.37	853–938	3	17.0 ± 2.6
Guizhou	Nayong	105.19, 26.81	1512–1571	5	25.3 ± 3.5
Guizhou	Sinan	108.11, 27.46	380–446	6	23.2 ± 4.2
Guizhou	Songtao	108.80, 28.08	389–451	6	27.0 ± 9.5
Guizhou	Yanhe	108.23, 28.30	470–516	6	23.9 ± 3.2
Guizhou	Zhenfeng	105.38, 25.20	939–1002	2	16.9 ± 0.4
Henan	Luoyang	112.24, 34.39	128	1	3.7 ± —
Hunan	Longshan	109.20, 29.14	343–455	6	22.8 ± 2.6
Hunan	Xiangtan	112.42, 27.51	64–74	2	20.5 ± 2.2
Hunan	Yiyang	112.17, 28.32	36–49	3	19.2 ± 5.4
Hunan	Yongzhou	111.46, 26.23	427	1	30.2 ± —
Sichuan	Baoxing	102.80, 30.21	948–1004	4	26.7 ± 6.5
Sichuan	Changning	104.79, 28.34	379–435	6	20.8 ± 6.8
Sichuan	Kaijiang	107.53, 31.04	425–449	4	26.2 ± 6.9
Sichuan	Lushan	102.56, 30.08	590–723	6	23.0 ± 5.0
Sichuan	Muchuan	103.53, 28.56	711–769	6	22.1 ± 3.9
Sichuan	Neijiang	105.13, 28.35	315–348	6	23.9 ± 7.0
Sichuan	Pengan	106.17, 30.83	281–295	6	30.9 ± 3.5
Sichuan	Pengzhou	103.50, 31.06	747–870	6	19.9 ± 6.6
Sichuan	Pingwu	104.49, 32.12	664–689	3	20.1 ± 0.9
Sichuan	Qingchuan	105.24, 32.37	553–594	6	30.8 ± 2.4
Sichuan	Qingshen	103.55, 29.49	392–449	6	32.8 ± 15.4
Sichuan	Santai	104.53, 30.82	380–389	6	19.0 ± 3.3
Sichuan	Tongjiang	107.25, 31.70	327–365	6	21.1 ± 6.5
Sichuan	Wangcang	106.39, 32.21	424–533	5	19.9 ± 3.9
Sichuan	Wusheng	106.28, 30.27	192–294	6	21.1 ± 5.9
Yunnan	Changning	99.41, 24.53	1699–1785	5	24.6 ± 4.1
Yunnan	Daguan	103.51, 27.47	1057–1308	4	24.0 ± 1.8
Yunnan	Honghe	102.25, 23.17	1379	1	29.8 ± —
Yunnan	Kunming	102.42, 25.03	1853	1	24.6 ± —
Yunnan	Shuangjiang	99.44, 23.26	1313–1328	2	26.8 ± 4.4
**Total (38 sites)**			37–1854	162	**23.3 ± 6.9**

### Morphological measurements

For each leaf, we measured: (i) leaf length (L, cm), from the base of the lamina to the apex along the midrib, with digital calipers (Mitutoyo CD-15CPX, Japan; precision 0.01 mm); (ii) maximum leaf width (W, cm), perpendicular to the midrib, with the same calipers; (iii) leaf thickness (LT, mm), averaged over three non-vein positions in the middle third of the lamina using a digital micrometer (Mitutoyo MDH-25 M, Japan; precision 0.001 mm); and (iv) leaf area (LA, cm²), measured on fresh leaves with a portable leaf-area meter (LI-3000 C, LI-COR Biosciences, Lincoln, NE, USA). The L/W ratio (dimensionless) was calculated as L / W for each leaf and then averaged at the clump and site level.

### Biomass and dry-matter traits

After morphological measurements, leaves were weighed for fresh mass (FW, g) using an analytical balance (Sartorius BSA224S, Germany; precision 0.1 mg), then oven-dried at 65 °C for 72 h until constant mass and re-weighed for dry mass (DW, g). SLA (cm² g⁻¹) was computed as LA / DW, and LDMC (%) as (DW / FW) × 100.

### Chlorophyll and photosynthetic performance

Leaf chlorophyll content was estimated with a SPAD-502Plus chlorophyll meter (Konica Minolta, Japan); three readings were taken along each leaf and averaged to yield a single SPAD value. Chlorophyll *a* fluorescence parameters were measured on fully dark-adapted leaves using a portable pulse-amplitude-modulated fluorometer (PAM-2500, Heinz Walz, Germany) after 30 min of dark adaptation with leaf clips. Measured parameters included minimum fluorescence (*F*o), maximum fluorescence (*F*m), variable fluorescence (*F*v = *F*m − *F*o), maximum photochemical efficiency of PSII (*F*v/*F*m), photochemical quenching coefficient (*q*P) and non-photochemical quenching (NPQ). Measurements were taken on cloud-free mornings between 08:00 and 11:00 local time.

### Leaf carbon, nitrogen and phosphorus

Dried leaves were ground to a fine powder using a ball mill (Retsch MM400, Germany) and passed through a 0.15 mm sieve. Total leaf carbon (TC, g kg⁻¹) and total leaf nitrogen (TN, g kg⁻¹) concentrations were determined by dry combustion on an elemental analyser (Vario MACRO cube, Elementar Analysensysteme GmbH, Langenselbold, Germany) using approximately 40 mg of leaf powder per sample. For leaf phosphorus (TP, g kg⁻¹), approximately 100 mg of ground sample was digested in a mixture of concentrated H₂SO₄ and H₂O₂, and P concentration in the digest was determined colorimetrically using the molybdenum-antimony-ascorbic acid method on an ultraviolet–visible spectrophotometer (UV-2600, Shimadzu, Japan) at 700 nm. Stoichiometric ratios C: N, N: P and C: P were calculated on a mass basis.

### Environmental variables

Geographic coordinates (longitude, latitude) and elevation were recorded in the field with a handheld GPS unit (Garmin GPSMAP 64s, USA; precision ± 3 m). Mean annual temperature (MAT, °C), mean annual precipitation (MAP, mm), maximum temperature of the warmest month and minimum temperature of the coldest month were extracted for each sampling coordinate from the WorldClim 2.1 global climate database at 30-arc-second (~ 1 km) resolution^40^, representing long-term climatic conditions averaged over 1970–2000. At each clump, topsoil samples (0–20 cm) were collected after removing the litter layer; soil water content (SWC, %) was determined gravimetrically after drying at 105 °C for 24 h, and soil bulk density (SBD, g cm⁻³) was measured using the cutting-ring method (volume 100 cm³). All environmental variables were assigned to site-level observations by averaging over clumps at each site.

### Statistical analysis

All analyses were conducted in Python 3.10 using *NumPy*, *SciPy*, *pandas*, *scikit-learn*, *statsmodels* and *matplotlib*. The significance threshold was α = 0.05. Variables were inspected for normality (Shapiro–Wilk test) and homogeneity of variance (Levene test) before parametric procedures.

To define L/W-based shape classes in a data-driven way, we applied *k*-means clustering to the L/W ratio of all 162 site-level observations for *k* = 2 to *k* = 6, with 10 random initialisations and a fixed random seed. The optimal *k* was selected using the silhouette coefficient, complemented by visual inspection of the L/W frequency distribution to ensure that the resulting classes were biologically interpretable. The resulting cluster boundaries are reported in the Results.

For leaf-area estimation we compared four candidate models on the complete site-level dataset: (i) a pooled Montgomery equation, LA = *k* · L · W, with a single proportionality coefficient fitted by least squares through the origin; (ii) a pooled multiple linear regression, LA = *a* + *b*₁L + *b*₂W; (iii) a class-specific Montgomery model with a separate *k* for each shape class; and (iv) a class-specific MLR with separate intercept and slope coefficients for each shape class. Including the class-specific Montgomery model alongside the pooled MLR allows us to disentangle the contribution of shape stratification from the contribution of the more flexible MLR functional form. Model performance was compared using the coefficient of determination (*R*²), root-mean-square error (RMSE) and Akaike information criterion (AIC).

Differences in functional traits among shape classes were tested by one-way ANOVA, and pairwise comparisons were performed using Tukey’s honestly significant difference (HSD) post-hoc test. Pairwise Pearson correlations were computed among all leaf traits. Conclusions focus on correlations that are both statistically significant and of non-trivial magnitude (|*r*| ≥ 0.30).

To assess the drivers of leaf functional trait variation, we proceeded in two steps. First, we computed pairwise Pearson correlations between each focal leaf trait (LA, L/W, LT, SLA, LDMC, TN, TP, SPAD, *F*v/*F*m, TC) and each environmental variable (elevation, MAT, MAP, SWC, SBD and latitude). Second, we fitted multiple linear regression models in which each focal trait was regressed on the full set of standardised environmental variables in order to estimate their joint and partial contributions; model fit was summarised by *R*² and overall *F*-test *p* value. Variables were standardised (mean = 0, SD = 1) prior to MLR so that regression coefficients are directly comparable in magnitude.

To assess the relative importance of within-site versus among-site variation, we partitioned the total sum of squares of each focal trait into within-site and among-site components based on a one-way ANOVA framework with site (county) as the grouping factor. The within-site sum of squares was the residual within-group SS, and the among-site sum of squares was the between-group SS; the percentage of total trait variance attributable to within-site variation was computed as 100 × SS_within / (SS_within + SS_among). Because climatic variables are identical for all observations within a given site, the within-site percentage represents an upper bound on the trait variation that is intrinsically unexplainable by among-site climate.

## Results

### Morphological variation and data-driven shape classification

Across the 162 site-level observations, leaf area averaged 23.3 ± 6.9 cm² (range 3.7–51.8 cm²; coefficient of variation [CV] = 29.6%), leaf length 15.2 ± 2.7 cm (CV = 17.9%), and leaf width 2.4 ± 0.4 cm (CV = 16.1%). The L/W ratio averaged 6.44 ± 0.98 (range 3.81–8.96, CV = 15.2%).

**Fig. 2:**
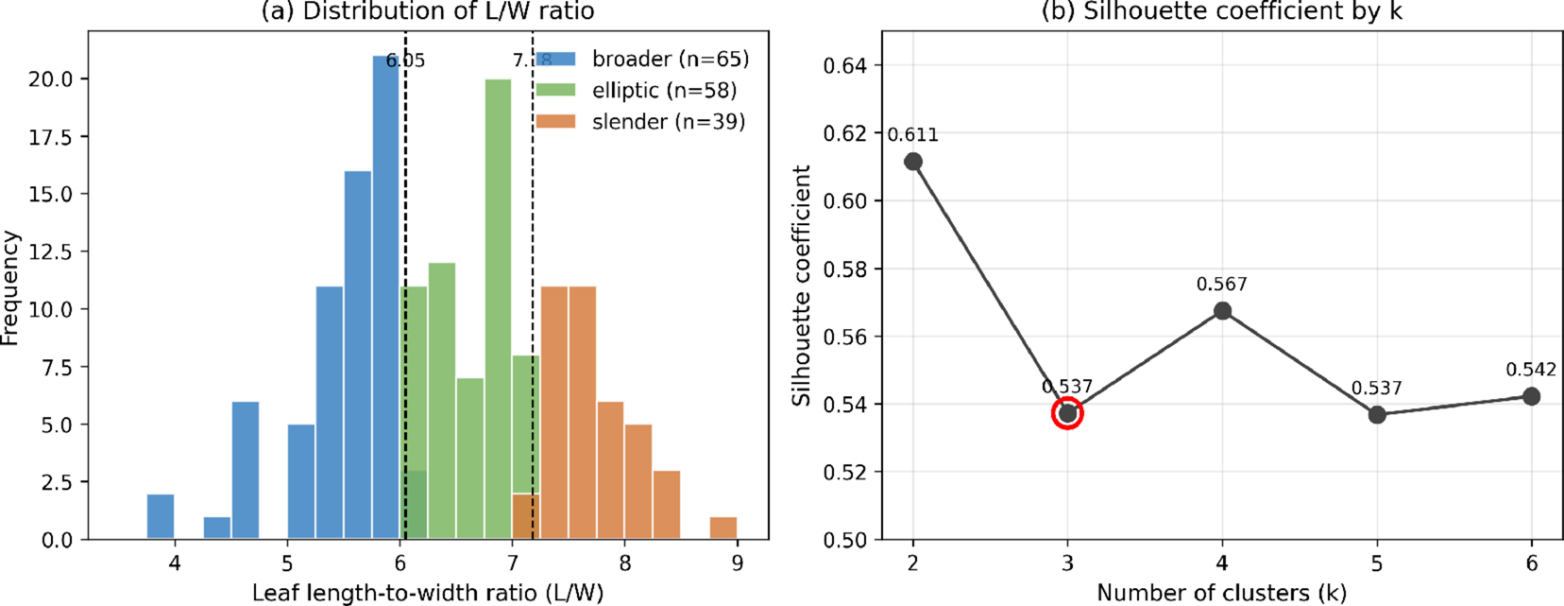
Data-driven classification of *N. affinis* leaves by length-to-width ratio. (**a**) Frequency distribution of L/W ratio across 162 site-level observations, coloured by the three k-means clusters (broader, elliptic, slender); vertical dashed lines mark the data-driven class boundaries at L/W = 6.05 and L/W = 7.18. (**b**) Silhouette coefficient as a function of the number of clusters k; k = 3 (circled in red) was retained as the optimal trade-off between statistical separation and biological interpretability.

*k*-means clustering of L/W ratio yielded silhouette coefficients of 0.611 at *k* = 2, 0.537 at *k* = 3, 0.567 at *k* = 4, 0.537 at *k* = 5 and 0.542 at *k* = 6 (Fig. 2b). The two-class solution split the distribution at L/W ≈ 6.5 into two contiguous halves; the three-class solution partitioned the data at L/W = 6.05 and L/W = 7.18 (Fig. 2a) and produced 65 broader (L/W ≤ 6.05), 58 elliptic (6.05 < L/W ≤ 7.18) and 39 slender (L/W > 7.18) observations. We adopted *k* = 3 in subsequent analyses.

### Leaf-area estimation: comparative model evaluation

**Fig. 3:**
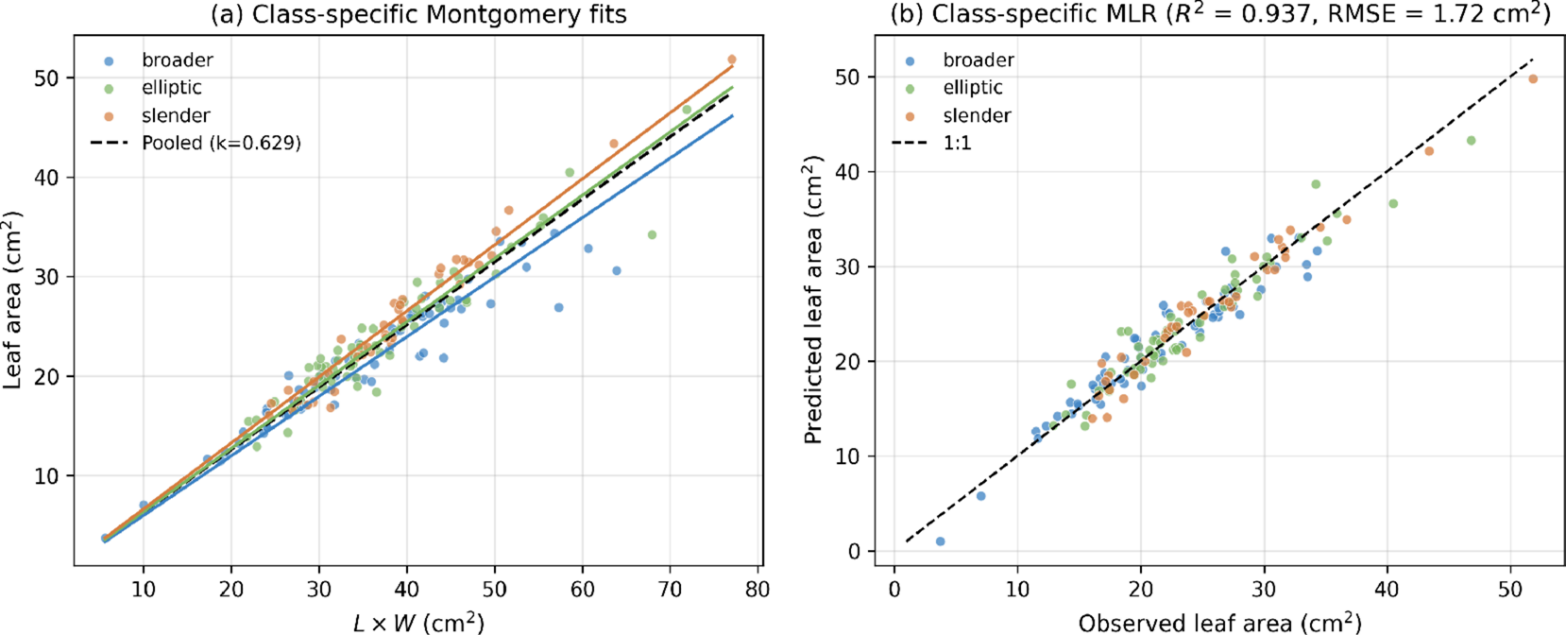
Leaf-area estimation performance. (**a**) Measured leaf area plotted against the product L × W for 162 site-level observations of *N. affinis*. The dashed black line shows the pooled Montgomery equation (LA = 0.629 · L · W), while coloured lines show class-specific Montgomery fits for broader, elliptic and slender leaves. The slope k increases systematically from 0.598 (broader) through 0.636 (elliptic) to 0.663 (slender). (**b**) Observed versus predicted leaf area for the best-performing class-specific multiple linear regression model (*R*² = 0.937, RMSE = 1.72 cm²). The dashed line is the 1:1 identity.

All four candidate models produced high-quality leaf-area predictions, but class-specific models showed lower RMSE and AIC than their pooled counterparts (Table 2; Fig. 3). The pooled Montgomery equation yielded *R*² = 0.899 and RMSE = 2.19 cm² with a single *k* = 0.629; the pooled MLR achieved a marginal improvement (*R*² = 0.905, RMSE = 2.13 cm², AIC = 252.5). Fitting the Montgomery equation separately within each of the three shape classes yielded a substantial improvement (*R*² = 0.920, RMSE = 1.95 cm², AIC = 224.5; ΔAIC = 34.0 relative to pooled Montgomery), with k increasing monotonically from broader (0.598) through elliptic (0.636) to slender leaves (0.663). The class-specific MLR achieved the best overall performance (*R*² = 0.937, RMSE = 1.72 cm², AIC = 196.6; ΔAIC = 27.9 relative to class-specific Montgomery).

**Table 2:** Comparison of four non-destructive leaf-area estimation models for *N. affinis* (*n* = 162 site-level observations). Lower AIC indicates better support; bold = best-performing model.

Model	*R*²	RMSE (cm²)	AIC	No. parameters
Pooled Montgomery (LA = k·L·W)	0.899	2.19	258.5	1
Pooled MLR (LA = a + b₁L + b₂W)	0.905	2.13	252.5	3
Class-specific Montgomery (one k per class)	0.920	1.95	224.5	3
**Class-specific MLR (a + b₁L + b₂W per class)**	**0.937**	**1.72**	**196.6**	**9**

### Functional trait variation across shape classes

Of the 14 functional traits examined, one-way ANOVA detected significant differences among shape classes for LA, L, W, LT, LDMC, TC, TN and TP (all *p* < 0.05; Table 3), whereas SLA (F = 1.80, *p* = 0.169), SPAD (F = 2.24, *p* = 0.110), *F*v/*F*m (F = 0.27, *p* = 0.764), and the C: N, N: P and C: P ratios did not differ significantly. Tukey HSD comparisons separated all three classes for L (broader < elliptic < slender) and partially separated the classes for LA, W, LT, LDMC, TC, TN and TP. LT was highest in slender leaves (0.106 ± 0.018 mm) and lowest in broader and elliptic leaves (both ≈ 0.099 mm). LDMC increased monotonically from broader (31.3 ± 9.5%) through elliptic (34.6 ± 6.7%) to slender (35.7 ± 8.6%) leaves. TC was highest in broader leaves (424.9 ± 21.8 g kg⁻¹) and lower in elliptic and slender leaves (≈ 414 g kg⁻¹). TN and TP were highest in broader leaves (28.8 ± 6.1 g kg⁻¹ and 3.23 ± 0.69 g kg⁻¹, respectively) and lowest in elliptic leaves (26.0 g kg⁻¹ and 2.88 g kg⁻¹). SLA, SPAD and *F*v/*F*m did not differ among classes.

**Table 3:** Functional trait variation across L/W-based shape classes in *N. affinis*. Values are means ± SD. Lowercase letters indicate significant differences among classes based on one-way ANOVA followed by Tukey HSD (*α* = 0.05); means sharing a letter are not significantly different.

Trait	Broader (*n* = 65)	Elliptic (*n* = 58)	Slender (*n* = 39)	F	*p*
Leaf area (cm²)	21.48 ± 6.40 a	23.79 ± 6.48 ab	25.73 ± 7.63 b	5.07	0.007
Leaf length (cm)	13.73 ± 2.66 a	15.58 ± 2.17 b	17.20 ± 2.09 c	27.27	< 0.001
Leaf width (cm)	2.50 ± 0.44 a	2.35 ± 0.32 ab	2.23 ± 0.31 b	6.84	0.001
Leaf thickness (mm)	0.099 ± 0.015 a	0.099 ± 0.015 ab	0.106 ± 0.018 b	5.01	0.008
SLA (cm² g⁻¹)	384.5 ± 151.5 a	361.7 ± 136.1 a	329.6 ± 139.5 a	1.80	0.169
LDMC (%)	31.33 ± 9.48 a	34.60 ± 6.74 ab	35.69 ± 8.64 b	4.01	0.020
SPAD	38.71 ± 4.29 a	39.52 ± 4.23 a	40.57 ± 4.61 a	2.24	0.110
*F*v/*F*m	0.77 ± 0.05 a	0.78 ± 0.09 a	0.78 ± 0.04 a	0.27	0.764
TC (g kg⁻¹)	424.9 ± 21.8 a	413.9 ± 22.2 b	414.2 ± 18.5 ab	5.14	0.007
TN (g kg⁻¹)	28.83 ± 6.11 a	25.95 ± 4.11 b	26.68 ± 4.45 ab	5.27	0.006
TP (g kg⁻¹)	3.23 ± 0.69 a	2.88 ± 0.41 b	3.07 ± 0.75 ab	4.83	0.009
C: N	15.29 ± 2.87 a	16.35 ± 2.77 a	15.97 ± 2.86 a	2.22	0.112
N: P	9.05 ± 1.38 a	9.10 ± 1.43 a	8.99 ± 1.91 a	0.06	0.939
C: P	137.4 ± 29.4 a	146.5 ± 21.0 a	141.1 ± 28.3 a	1.86	0.159

### Trait coordination

**Fig. 4:**
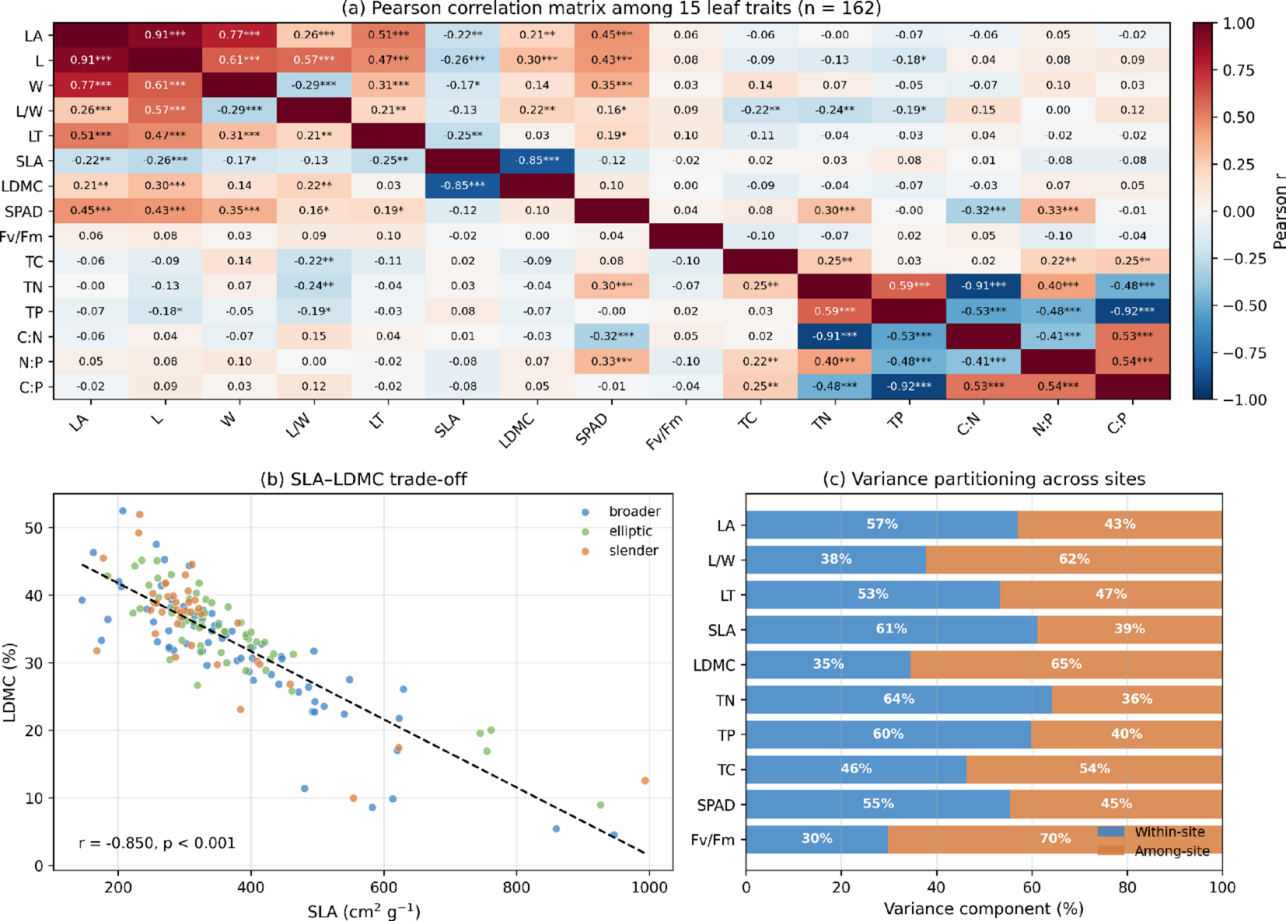
Trait coordination, the SLA–LDMC trade-off and variance partitioning in *N. affinis*. (**a**) Pearson correlation matrix among 15 leaf morphological, structural and chemical traits (*n* = 162). Numerical values are Pearson r coefficients; asterisks denote significance (*p < 0.05, **p < 0.01, ***p < 0.001). (**b**) SLA–LDMC trade-off plotted by shape class; the dashed line is the linear regression fit to all points. (**c**) Variance partitioning of ten focal traits between within-site and among-site components based on a one-way ANOVA framework with site as the grouping factor.

Pairwise Pearson correlations among leaf traits (Fig. 4a) showed a structured coordination network. The strongest single correlation was between SLA and LDMC (*r* = − 0.850, *p* < 0.001; Fig. 4b). Leaf N and P concentrations were positively correlated (*r* = 0.590, *p* < 0.001). Leaf C: N was strongly negatively correlated with TN (*r* = − 0.91, *p* < 0.001), and C: P with TP (*r* = − 0.92, *p* < 0.001).

Leaf area was uncorrelated with TN (*r* = − 0.003, *p* = 0.97) and TP (*r* = − 0.07, *p* = 0.38), and only weakly related to SLA (*r* = − 0.22, *p* = 0.004) and LDMC (*r* = 0.21, *p* = 0.007). The L/W ratio correlated weakly with LES traits: with SLA (*r* = − 0.13, *p* = 0.091), LDMC (*r* = 0.22, *p* = 0.005), TN (*r* = − 0.24, *p* = 0.002) and TP (*r* = − 0.19, *p* = 0.018).

### Environmental drivers and variance partitioning

Bivariate correlations between environmental variables and leaf traits were generally weak, with all |*r*| ≤ 0.30 (Table 4). The strongest associations were with soil bulk density: SBD correlated positively with LT (*r* = + 0.30, *p* < 0.001), LA (*r* = + 0.20, *p* = 0.011), SLA (*r* = + 0.17, *p* = 0.037) and TP (*r* = + 0.17, *p* = 0.030), and negatively with LDMC (*r* = − 0.25, *p* = 0.001) and L/W ratio (*r* = − 0.23, *p* = 0.004). LT was also weakly negatively correlated with elevation (*r* = − 0.16, *p* = 0.038). MAT, MAP, SWC and latitude showed no significant bivariate relationships with most traits. Multiple linear regressions of each trait on the full set of environmental variables yielded explained variances of *R*² = 0.05–0.15 (Table 4); regressions for L/W (*R*² = 0.145, *p* < 0.001), LA (*R*² = 0.137, *p* < 0.001), LDMC (*R*² = 0.128, *p* = 0.002) and LT (*R*² = 0.120, *p* = 0.003) were significant overall.

Variance partitioning of trait variation between within-site and among-site components (Table 5; Fig. 4c) showed that within-site variation was substantial across all focal traits. The within-site fraction was 64% for TN, 61% for SLA, 60% for TP, 57% for LA, 55% for SPAD, 53% for LT and 46% for TC. For LDMC and L/W, the among-site fraction was larger (65% and 62%, respectively), and for *F*v/*F*m the among-site fraction was 70%. Across the ten focal traits, the within-site fraction averaged 51%.

**Table 4:** Pearson correlations between environmental variables and key leaf functional traits of *N. affinis* (*n* = 162; one observation lacks elevation). Multiple linear regression *R*² is shown for each trait regressed on all environmental variables jointly. MAT = mean annual temperature, MAP = mean annual precipitation, SWC = soil water content, SBD = soil bulk density. *p < 0.05; **p < 0.01; ***p < 0.001.

Trait	Elevation	MAT	MAP	SWC	SBD	Latitude	MLR *R*²
LA	+ 0.01	+ 0.05	+ 0.17*	−0.09	+ 0.20*	+ 0.04	0.137***
L/W	−0.06	−0.10	+ 0.12	+ 0.04	−0.23**	−0.06	0.145***
LT	−0.16*	+ 0.06	+ 0.09	−0.09	+ 0.30***	+ 0.06	0.120**
SLA	+ 0.05	−0.14	−0.07	+ 0.10	+ 0.17*	−0.04	0.066
LDMC	−0.05	+ 0.06	+ 0.06	−0.03	−0.25**	+ 0.11	0.128**
TN	−0.03	+ 0.15	−0.01	−0.15	−0.08	+ 0.00	0.040
TP	+ 0.01	−0.07	−0.10	+ 0.08	+ 0.17*	+ 0.11	0.046

**Table 5:** Variance partitioning of leaf functional traits in *N. affinis* between within-site and among-site components (*n* = 162 site-level observations across 38 county-level sites). The total sum of squares was decomposed via a one-way ANOVA framework with site as the grouping factor; values are percentages. Traits are ordered by descending within-site percentage.

Trait	Within-site variation (%)	Among-site variation (%)
Leaf TN	64.2	35.8
SLA	61.1	38.9
Leaf TP	59.8	40.2
Leaf area (LA)	57.1	42.9
SPAD	55.5	44.5
Leaf thickness	53.3	46.7
Leaf TC	46.3	53.7
L/W ratio	37.9	62.1
LDMC	34.6	65.4
*F*v/*F*m	29.8	70.2

## Discussion

### Shape-specific leaf-area estimation improves non-destructive assessment

Stratifying *N. affinis* leaves by L/W-based shape class before fitting substantially improved non-destructive leaf-area estimation. The Montgomery proportionality coefficient k increased monotonically from broader (0.598) through elliptic (0.636) to slender leaves (0.663), an 11% rise across the observed L/W range. Because k is the fraction of the bounding rectangle occupied by the lamina, this monotonic increase has a simple geometric interpretation: as leaves become more elongated, the relative contribution of the narrowing base and apex shrinks and the lamina more closely approximates a parallel-sided strip with k approaching the limiting value of approximately 2/3 for a true ellipse. This pattern is consistent with geometric predictions and empirical work on leaf allometry^5,7–11,23,24^. The class-specific MLR outperformed both pooled models and the class-specific Montgomery model (AIC 196.6 vs. 252.5 for pooled MLR and 224.5 for class-specific Montgomery), indicating that both stratification and the inclusion of separate linear effects of L and W contributed to predictive accuracy. The improvement of the class-specific Montgomery model over the pooled Montgomery model also indicates that shape stratification itself contributed to model performance by allowing k to vary with lamina geometry. From a practical standpoint, the class-specific MLR provides the best compromise between accuracy and parsimony, whereas the simpler class-specific Montgomery model remains useful when field calculations require only one coefficient per shape class.

### Partial correspondence between leaf shape and the intraspecific LES

Our second major finding is that L/W-based shape classes capture part — but not all — of the intraspecific LES in *N. affinis*. Traits most closely associated with tissue density and construction cost (LT, LDMC and TC) differed significantly among classes, with slender leaves tending to be thicker and to have higher LDMC, consistent with a more slow-return investment strategy^2,14,15,33–35^. Leaf N and P concentrations also differed significantly, with broader leaves showing higher TN and TP than elliptic and slender leaves, in the direction expected if broader leaves operate closer to a fast-return strategy^1,2,41^. The strong SLA–LDMC trade-off we detected (*r* = − 0.850) is consistent with the global LES^1,2,14,15^, and its recovery within a single species across a ~ 2,000 km × 1,800 m geographic and elevational range provides evidence that key LES trade-offs can also be expressed within species, including in woody bamboos^17–19,37,38^.

Critically, however, three LES-central traits — SLA, SPAD chlorophyll index and *F*v/*F*m — did not differ significantly among shape classes, and L/W ratio correlated only weakly (|*r*| < 0.25) with these traits when analysed continuously. This partial decoupling has two implications. First, leaf shape in *N. affinis* is a reliable marker of structural and dry-matter-related LES traits but is a comparatively poor marker of SLA and short-term photosynthetic performance, which appear to vary along a partly independent axis. Second, the observed SLA–LDMC trade-off is driven primarily by variation in dry-matter density (captured by LDMC and class) rather than by variation in leaf thinness per unit dry mass (captured by SLA), suggesting that in this species the rate-limiting axis of the intraspecific LES is dry-matter allocation rather than biomass-per-area. The pattern is therefore more nuanced than a simple “broader = fast, slender = slow” dichotomy: L/W-based classes mark a structural–chemical gradient, but not a full photosynthetic gradient.

### Leaf size and chemistry vary on partly independent axes

Variance partitioning and the pairwise correlations together indicate that within *N. affinis*, leaf area and leaf chemistry vary on partly independent axes. Leaf area was uncorrelated with TN and TP (|*r*| < 0.07) and only weakly related to SLA and LDMC (|*r*| ≈ 0.22), whereas the SLA–LDMC trade-off across all 162 observations was extremely strong. This pattern contrasts with the broader interspecific LES, in which leaf size, SLA and N tend to covary^1,2,13,14^, and is consistent with a growing body of work showing that intraspecific trait variation is organised along fewer and different axes than interspecific variation^16–19^. A practical consequence is that leaf size and leaf chemical composition need to be measured and modelled independently in bamboo ecosystem applications.

### Local heterogeneity outweighs broad climatic signals

Environmental variables explained limited variation in our trait data: no single bivariate correlation exceeded |*r*| = 0.30, and multiple regressions of focal traits on the full environmental set yielded *R*² ≤ 0.15. The largest joint signals occurred for L/W, LA, LDMC and LT, and soil bulk density was the most frequently significant individual environmental variable. Variance partitioning (Table 5; Fig. 4c) clarifies the scale dependence of these weak environmental relationships. Of the ten focal traits, six (TN, SLA, TP, LA, SPAD and LT) had within-site fractions of 53–64%, meaning that more than half of their total variance occurred at scales finer than the county-level sampling site. Because broad climatic variables were assigned at the site level, this within-site component cannot be captured by macroclimatic predictors. Conversely, LDMC, L/W and *F*v/*F*m showed larger among-site components, suggesting that some traits may still respond to broader site-level differences, although the climatic variables used here captured only a small fraction of that variation. These results support a nuanced interpretation: broad climate is a weak predictor of *N. affinis* leaf traits relative to local heterogeneity, rather than climate being irrelevant to leaf functional variation^16–19^.

### Implications, caveats and future directions

The L/W-based classification framework provides a practical, interpretable tool for non-destructive leaf-area estimation in *N. affinis* (class-specific MLR *R*² = 0.937), and clarifies which parts of the intraspecific LES are tracked by leaf shape. Four caveats apply: (i) analyses were conducted on site-level means rather than on individual leaves, so within-clump variation is not addressed; (ii) sampling covered seven provinces but not the species’ full range; (iii) the cross-sectional design cannot separate genetic from plastic components or establish causality; and (iv) sampling was restricted to the growing season. The most informative future step would combine common-garden experiments with anatomical (vein density, mesophyll thickness, stomatal traits) and high-resolution photosynthetic measurements to resolve whether the partial decoupling of SLA and photosynthetic performance from shape is intrinsic to the species’ trait architecture.

## Conclusions

Three conclusions emerge from this study of *N. affinis* across southern China. (1) Classifying leaves into data-driven broader, elliptic and slender shape classes by L/W ratio improves non-destructive leaf-area estimation relative to pooled Montgomery and pooled MLR models; the Montgomery proportionality coefficient k increases monotonically from broader (0.598) through elliptic (0.636) to slender leaves (0.663), and a class-specific MLR provides the best overall predictive performance (*R*² = 0.937, RMSE = 1.72 cm²). (2) L/W-based shape classes capture significant variation in leaf thickness, dry-matter content and leaf C, N and P concentrations, and a strong SLA–LDMC trade-off operates at the intraspecific level, demonstrating that part of the leaf economics spectrum is recoverable from a simple morphological descriptor. However, SLA, SPAD and Fv/Fm do not differ among classes, showing that leaf shape in *N. affinis* tracks structural and dry-matter axes of the LES but is largely decoupled from SLA and from short-term photosynthetic performance. (3) Environmental variables explain limited trait variation; multiple-regression *R*² values are ≤ 0.15, and 30–64% of trait variance occurs within sites, indicating that a substantial share of variation lies below the broad climatic scale captured here. The combined morphological and functional framework provides a practical tool for bamboo ecosystem assessment and a nuanced view of how the LES manifests within a single morphologically plastic species.

## Data Availability

The complete dataset (*n* = 162 site-level functional trait observations from 38 sites) is available from the corresponding author upon reasonable request and will be deposited in a public repository upon acceptance.
